# Mid-Term Outcomes of Isolated Trochlear Arthroplasty for Patellofemoral Arthritis: A Joint-Preserving Alternative to Complete Patellofemoral Replacement

**DOI:** 10.7759/cureus.104473

**Published:** 2026-03-01

**Authors:** Sivashankaran Munuswamy, Kamparsh Thakur, Rohan Bassi, Varun Vig, Divya Prakash, Vishal Paringe

**Affiliations:** 1 Trauma and Orthopaedics, Sandwell and West Birmingham NHS Trust, Birmingham, GBR

**Keywords:** functional outcomes, implant survivorship, kujala score, patellofemoral arthritis, trochlear arthroplasty

## Abstract

Background

Trochlea-only arthroplasty (TOA) is a conservative surgical approach for isolated patellofemoral arthritis, aimed at preserving the native patella and avoiding complications associated with its resurfacing. This study evaluates the mid-term survivorship, functional outcomes, and revision profile of patients undergoing TOA and compares outcomes with historical data from conventional patellofemoral arthroplasty (PFA).

Methods

We retrospectively reviewed 50 patients who underwent isolated TOA between 2008 and 2019 by a single surgeon. A medial parapatellar approach was used, with intraoperative assessment of the joint. The decision to retain the native patella was made irrespective of the extent of arthritis in the patella. All patients underwent patelloplasty and circum-patellar neurectomy without resurfacing. Implant survival was evaluated using Kaplan-Meier analysis, and factors influencing revision were assessed using Cox proportional hazards modelling. Patient-reported outcomes were collected using the Kujala score in a subset of patients with unrevised implants.

Results

The overall implant survivorship was 82% at a mean follow-up of 9.2 years. Nine (18%) patients required revision, most commonly due to progression of tibiofemoral arthritis. Revisions included patellar resurfacing, staged medial unicompartmental knee replacement, and conversion to total knee arthroplasty. Age and sex were not significant predictors of revision. The mean Kujala score in 13 (32%) unrevised patients was 56.1, with no reports of instability or significant flexion deficits. No patients with known revisions were contacted for patient-reported outcomes.

Conclusion

TOA is a viable joint-preserving surgical option for selected patients with isolated patellofemoral arthritis. It avoids the complications associated with patellar resurfacing and maintains satisfactory mid-term survivorship comparable to published literature. TOA may serve as a procedural bridge between conservative management and full PFA, allowing for a stepwise and anatomically conservative approach. Further prospective studies are warranted to validate these findings.

## Introduction

Proximal Isolated patellofemoral joint (PFJ) arthroplasty is indicated in patients with symptomatic PFJ arthritis, preserved medial and lateral tibiofemoral compartments, and predominant anterior knee pain. The prevalence of isolated PFJ arthritis is reported in 8% of women and 2% of men over the age of 55 years [1].

Although total knee arthroplasty (TKA) has been used for advanced PFJ arthritis, it is frequently considered overly invasive in such cases. TKA sacrifices unaffected compartments, disrupts native biomechanics, and poses long-term implications, especially in younger patients [2]. In contrast, patellofemoral arthroplasty (PFA) offers comparable pain relief with reduced soft tissue trauma, lower blood loss, fewer perioperative complications, and a faster recovery [3]. Revision of PFA to TKA is feasible with generally satisfactory outcomes [4]. 

Traditional PFA involves resurfacing both the trochlea and the patella. The trochlea is reconstructed using a cemented metallic component, while the patella receives an all-polyethylene button. However, patellar resurfacing is associated with several complications including patellar fracture, polyethylene wear, aseptic loosening, patellar osteonecrosis, and instability [5]. These risks are more pronounced in patients with smaller patellae, such as those of Asian descent [6]. Lateral retinacular release, occasionally necessary to improve tracking, further reduces vascularity and may increase fracture risk [7]. Additionally, thermal necrosis during cement polymerisation and a 30% to 40% increase in strain have been observed in resurfaced patellae, contributing to extensor mechanism compromise [8]. 

The engagement of the patella within the trochlear groove varies systematically with knee flexion. In full extension (0°), the patella lies proximal to the trochlea with minimal bony contact. As flexion begins (10°-30°), the inferior pole of the patella starts to articulate with the proximal trochlea, initiating engagement. Between 30° and 60°, the patella becomes progressively seated within the groove, with increasing bony congruency and contact area. In deeper flexion (60°-90°), engagement deepens further and contact shifts proximally on the patella and distally on the femur [9]. Beyond this, it exits the trochlear groove and articulates with the femoral intercondylar notch. This detail underscores the limited mechanical relevance of the PFJ interface in deep flexion, suggesting that trochlear-only resurfacing may be functionally adequate in cases of patellofemoral arthritis.

The rationale for isolated trochlear resurfacing is conceptually similar to hemiarthroplasty in femoral neck fractures, where only the affected femoral head is replaced to preserve uninvolved anatomy and minimise complications. [10]. Retaining the native patella may avoid complications associated with patellar resurfacing, preserve extensor mechanism integrity, and simplify future revision surgery if required.

While conventional PFA has shown short- to mid-term results comparable to TKA [11], to our knowledge, no prior studies have specifically assessed the clinical outcomes or survivorship of isolated trochlear arthroplasty. This study aims to evaluate patient-reported outcomes, satisfaction, and implant survival following isolated trochlear resurfacing, using revision surgery as the primary endpoint.

## Materials and methods

Study design

The study was registered as an audit with the Trust Audit Module Database (via SafeGuard Webportal by Ulysses). Ethical clearance was obtained from Sandwell and West Birmingham Hospitals NHS Trust, Birmingham, United Kingdom. The NHS Research Ethics Committee decision tool excluded the need for further ethical approval. This study was a retrospective survival analysis of patients who underwent isolated trochlear-only arthroplasty (TOA) for symptomatic patellofemoral arthritis. The inclusion criteria incorporated all adults who had undergone isolated TOA in any sector. Body mass index (BMI) and comorbidities were not taken into consideration. All procedures were performed by a single fellowship-trained orthopaedic surgeon between 2008 and 2019. The primary endpoint was defined as prosthesis revision for any reason. Patient death and viable implants at final follow-up were considered censored observations.

Surgical technique

A standard medial parapatellar approach was used in all cases. Patellar resurfacing was not performed routinely, as long as the patella was found to track satisfactorily in the trial trochlear implant. Nevertheless, all patients underwent patellar osteophyte removal, patelloplasty, and circum-patellar neurectomy to reduce pain from impingement of the osteophytes against the soft tissues and reduce anterior knee pain. No intraoperative complications or conversions to TKA occurred in any of the cases.

Data collection

The study population was identified from prospectively maintained records within the surgeon’s National Joint Registry (NJR) submissions and from the hospital records. Demographic data, including age at surgery and sex, were collected, along with clinical outcomes including revision, death, and implant viability. Postoperative follow-up data included implant survival time in years. Additionally, patients with unrevised implants were contacted, and the follow-up Kujala Anterior Knee Pain Score was calculated. The Kujala score evaluates pain, functional ability, swelling, abnormal patellar tracking, muscle atrophy, and range-of-motion deficits [12]. Each patient was contacted by telephone by a clinician who had no prior involvement in any of the arthroplasty procedures. The original operating team did not participate in data collection, helping to minimise potential interviewer bias in the study. Patients with known revision procedures were not contacted in order to avoid response bias and because their clinical course had already diverged from the primary intervention pathway.

Statistical analysis

A Kaplan-Meier analysis was conducted to estimate prosthesis survival with additional stratification by age group. Differences in survival functions between age groups were assessed using the log-rank test. A Cox proportional hazards model was employed to determine predictors of revision, with covariates including age (continuous), age group (categorical), and sex. A p-value less than 0.05 was considered statistically significant.

## Results

A total of 50 (consecutive) patients underwent TOA for patellofemoral arthritis between 2008 and 2019. A total of 46 (92%) patients were female, and the rest (8%) were male. The mean age at the time of surgery was 55.7 years (range: 42-78). The outcomes were classified into three endpoints: implant viability, revision, or patient death.

At final follow-up, 41 (82%) implants remained viable. Nine patients (18%) underwent revision surgery, with a mean time to revision of 3.8 years. There were four (8%) reported deaths unrelated to the index surgery. The mean follow-up duration for patients with unrevised implants was 9.2 years. Revision procedures included patellar resurfacing, staged addition of a medial unicompartmental knee replacement (UKR), or conversion to TKA.

Survival analysis

**Figure 1:**
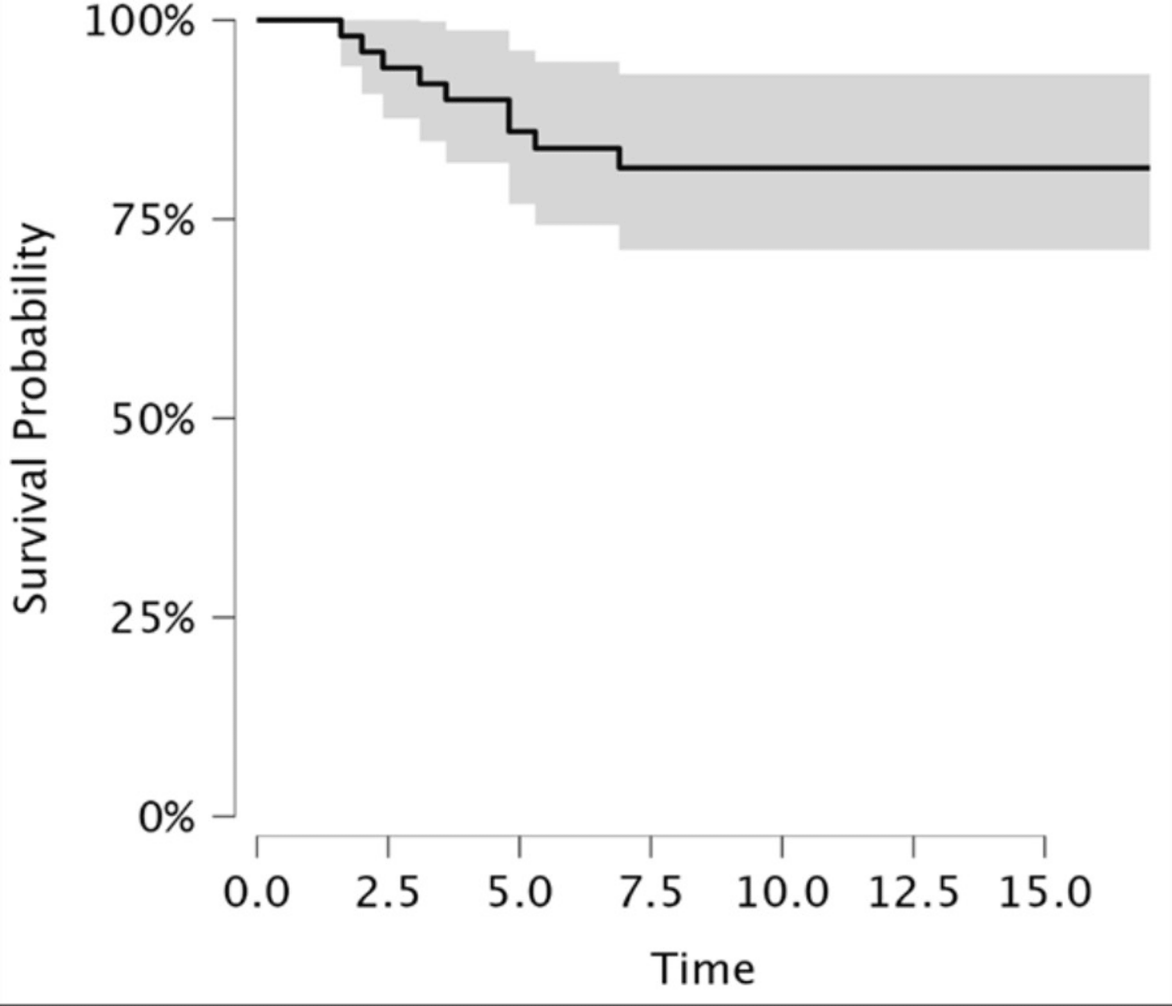
Kaplan-Meier curve showing time of overall survival of prosthesis in years.

**Figure 2:**
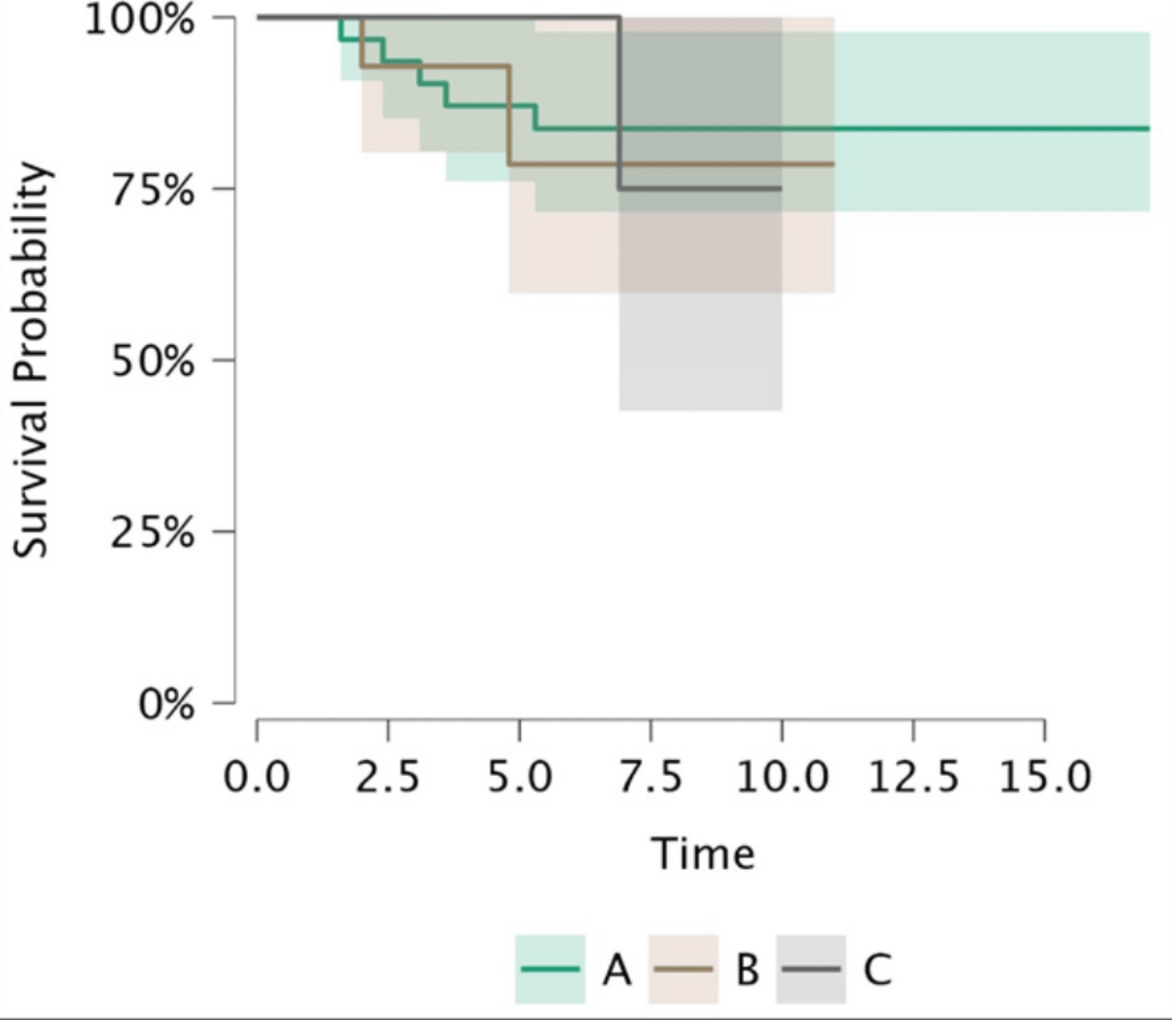
Kaplan-Meier curves for implant survival in years stratified by age group. Group A: <60 years; Group B: 60-70 years; Group C: >70 years

The Kaplan-Meier survival estimate demonstrated an overall prosthesis survival rate of 82% at final follow-up (Figure 1). When stratified by age group-Group A (<60 years), Group B (60-70 years), and Group C (>70 years)-no statistically significant difference in survival was observed (using log-rank test, p = 0.91) (Figure 2). The restricted mean survival time was similar across all three age strata (Table 1).

**Table 1:** Total numbers and revision frequencies distributed by age group with corresponding restricted mean survival data.

Age group (years)	Total number, n (%)	Revision frequency, n (%)	Restricted Mean Survival (years)	Standard Error
Group A (<60)	31 (62%)	5 (10%)	14.760	0.921
Group B (60-70)	14 (28%)	3 (6%)	14.186	1.449
Group C (>70)	5 (10%)	1 (2%)	14.475	2.187

Cox proportional hazards analysis

The Cox model revealed no statistically significant predictors of prosthesis revision (Table 2). This analysis was performed on only nine revision patients, which makes this analysis underpowered and the findings not absolutely consistent. Age at surgery (hazard ratio (HR) 1.02, 95% CI 0.89-1.16; p = 0.80) and male sex (HR 1.63, 95% CI 0.18-15.09; p = 0.67) were not associated with increased risk. Age groups B and C also did not differ significantly from Group A in terms of HRs. 

**Table 2:** Cox proportional hazards summary for covariates of age at surgery, age group and sex. The Cox hazard coefficient is given with corresponding hazard ratios (HR) and significance p-values.

Variable	Cox Hazard coefficient	Standard Error	HR	95% CI	p-value	Interpretation
Age at Surgery	0.020	0.067	1.02	0.89 – 1.16	0.80	No significant effect of age on revision risk.
Sex (Male)	0.489	1.130	1.63	0.18 – 15.09	0.67	Males had a higher hazards ratio, but not statistically significant.
Age Group B	-0.061	1.410	0.94	0.06 – 15.24	0.96	No significant difference from Group A.
Age Group C	-0.301	2.130	0.74	0.01 – 42.81	0.89	No significant difference from Group A.

Patient-reported outcomes

Thirteen patients with unrevised implants (32%) were successfully contacted via telephone for outcome assessment using the Kujala Anterior Knee Pain Score. Limited contact was attributed to patients who underwent surgery in the private sector. The mean Kujala score was 56.1 (range: 37-85) out of 100. No patients reported painful patellar tracking or instability following TOA. All respondents reported either a full range of flexion or only a minor deficit. None experienced severe flexion limitation.

## Discussion

Study rationale and avoiding risks of patellar resurfacing

PFA is an established surgical treatment for isolated PFJ arthritis and has shown encouraging mid- to long-term results with modern implant designs [13]. However, isolated TOA remains less explored and underreported. This study presents mid-term implant survivorship, complication profile, and patient-reported outcomes in a cohort undergoing TOA, with a particular emphasis on the rationale for avoiding primary patellar resurfacing.

Early-generation PFA implants, including the Richard’s prosthesis, were associated with high failure rates due to factors such as component malposition, polyethylene wear, non-anatomic design, and patellar maltracking [14]. Second-generation implants introduced in the 1990s addressed these limitations through improved design and anatomical conformity, thereby enhancing patellar tracking and reducing instability [3]. Despite advances in implant design, debate continues over the necessity of routine patellar resurfacing during PFA. Notably, the orthopaedic community has increasingly questioned the role of patellar resurfacing in TKA, citing limited benefits and potential risks [15].

Primary resurfacing of the patella may lead to complications such as fracture, component loosening, anterior impingement, and maltracking. This particularly occurs when the prosthetic patellar button is misaligned or demonstrates curvature mismatch with the prosthetic trochlea [7]. In this series, no cases of patellar maltracking or instability were encountered, possibly suggesting that articulation between the native patella and prosthetic trochlea may be functionally effective. Furthermore, at flexion angles beyond 90 degrees, the patella typically disengages from the trochlear groove and articulates with the distal condyles of the native femur. This mismatch may lead to discomfort when a polyethylene patella contacts the native joint surface, justifying patellar preservation in appropriate cases [16].

Preserving the patella in TKA

Evidence from TKA literature further supports this approach. Omitting patellar resurfacing during TKA has been shown to yield comparable functional outcomes while potentially reducing the risk of patellar-specific complications. Several studies have demonstrated no significant difference in knee scores, patient satisfaction, or implant survivorship between resurfaced and non-resurfaced groups. He et al. (2011) [17] and Pavlou et al. (2011) [18] reported in meta-analyses that non-resurfacing does not lead to inferior outcomes and avoids risks such as patellar fracture, avascular necrosis, or component loosening. By extension, the rationale may be applied to PFA: avoiding routine resurfacing may reduce risk without compromising outcomes. Additionally, TOA potentially preserves future surgical options. If symptoms recur, staged patellar resurfacing remains feasible without necessitating full conversion to TKA. Schiavone Panni et al., in a meta-analysis of TKA outcomes, also found no added benefit with primary patellar resurfacing. However, they highlighted associated risks including fracture, extensor mechanism disruption, wear, and loosening [19].

The role of patellar tracking is critical in determining outcomes of PFA. Argenson et al. identified maltracking as a leading cause of failure in PFJ replacement, advocating for meticulous component positioning and soft-tissue balancing [11]. van der List et al. highlighted patellar maltracking as one of the major causes behind revision of the PFA [20]. These findings validate our intraoperative strategy to some extent, where patellar tracking (using no thumb technique), not cartilage loss, served as the principal criterion for proceeding with TOA. In our limited experience, even patients with significant patellar cartilage wear may be considered suitable for TOA if tracking remains congruent.

Implant survivorship

Our cohort demonstrated an overall prosthesis survival rate of 82% at a mean follow-up of 9.2 years, which is consistent with published mid-term data. A systematic review of international joint registries reported a mean 10-year survival of 82.23% (95% CI 78.90-85.56) for conventional PFA, aligning closely with our findings [21]. A systematic review by van der List et al. reported 5-, 10-, 15-, and 20-year survival rates for PFA of 91.7%, 83.3%, 74.9%, and 66.6%, respectively [22]. Similar mid-term outcomes have been observed in single-centre studies by Kooijman et al. [23]. Within the limits of the study, these results suggest that TOA can achieve comparable durability to full PFJ replacement without exposing the patient to risks of patellar resurfacing.

Patient-reported outcomes

Patient-reported outcomes were evaluated using the Kujala Anterior Knee Pain Score. This is a validated scoring system for patients with patellofemoral pain and is free to use with no licensing requirements [24]. The mean score was 56.1, which reflects reasonable function given the complex pathology. Ajnin et al reported comparable patient-reported outcomes at five-year follow-up in patients undergoing conventional PFA [25]. However, these findings can't be generalized or compared functionally with the previously published outcome studies for conventional PFA, as the number of patients with their outcomes reported is fairly small. Importantly, none of our patients experienced patellar dislocation or significant restriction in range of motion.

Joint-preservation rationale for TOA

Revision was required in 18% of cases; the reason is unclear as to whether this was due to the natural progression of tibiofemoral arthritis or inappropriate patient selection in the first place. Notably, revision strategies varied and included secondary patellar resurfacing, addition of medial unicompartmental knee arthroplasty (UKA), or conversion to TKA. The ability to perform staged resurfacing of the patella is a unique feature of TOA. This flexibility reinforces TOA as a joint-preserving and stepwise approach, facilitating tailored management of PFJ arthritis. Age and sex were not found to be statistically significant predictors of revision risk, although younger patients exhibited a non-significant trend toward higher revision rates, likely due to greater functional demands and longer implant exposure [26]. Again, these findings can't be generalised as the Cox hazards model analysis was slightly underpowered in this scenario.

This study reinforces the rationale for a selective, anatomy-preserving approach to isolated PFJ arthritis. Intraoperative assessment of patellar tracking, rather than cartilage integrity alone, should guide surgical decision-making. This suggests that functional tracking, along with cartilage loss, rather than radiographic or intraoperative cartilage grading alone, may be the more critical determinant in selecting patients for trochlear-only resurfacing. Advanced imaging modalities, such as MRI or dynamic CT scan aids in identifying maltracking preoperatively and may assist in excluding cases requiring full patellofemoral arthroplasty.

Study limitations

This study has several limitations inherent to its retrospective design. First, the sample size is relatively small (n = 50), which limits the power of subgroup analyses, particularly across age and sex strata. Second, the number of patients available for follow-up using validated patient-reported outcome measures (PROMs) was restricted, primarily due to contact challenges and patients having undergone their index procedures in the private sector. Third, there was no control arm of patients undergoing conventional PFA with patellar resurfacing, which limits direct comparative conclusions. Additionally, functional outcome was assessed using a single PROM score, which, although validated, may not capture all aspects of knee-related quality of life. Finally, this is a single-surgeon series, which, while ensuring consistency in technique and enabling a degree of internal validity, may limit generalisability to broader surgical practice.

## Conclusions

Isolated TOA offers a viable, anatomically conservative solution for the management of isolated patellofemoral arthritis in appropriately selected patients. This approach preserves the native patella, avoids complications associated with resurfacing, and provides acceptable mid-term survivorship. In the absence of maltracking, primary patellar resurfacing may not be absolutely necessary.

Importantly, TOA may be conceptualised as a bridging procedure that lies between conservative management and complete PFJ arthroplasty. It allows for symptomatic relief while preserving native structures and leaving open the possibility of staged intervention, such as patellar resurfacing or conversion to TKA. This stepwise approach supports joint preservation principles, particularly in younger and more active patients. Further prospective, comparative studies with larger cohorts and long-term follow-up are warranted to validate these findings and define optimal indications.
